# *Akkermansia muciniphila* Colonization Alleviating High Fructose and Restraint Stress-Induced Jejunal Mucosal Barrier Disruption

**DOI:** 10.3390/nu14153164

**Published:** 2022-07-30

**Authors:** Jiayu Yu, Tianlong Liu, Zihao Gao, Runbang Liu, Zixu Wang, Yaoxing Chen, Jing Cao, Yulan Dong

**Affiliations:** College of Veterinary Medicine, China Agricultural University, Beijing 100193, China; yujy7288338@163.com (J.Y.); liutianlong@cau.edu.cn (T.L.); gaozihao@cau.edu.cn (Z.G.); lrb@cau.edu.cn (R.L.); zxwang2007@163.com (Z.W.); yxchen@cau.edu.cn (Y.C.); caojing315@126.com (J.C.)

**Keywords:** *Akkermansia muciniphila*, high fructose diet and restraint stress, Paneth cell, NLRP6, autophagy

## Abstract

*Akkermansia muciniphila* (*A. muciniphila*) is a mucin-degrading bacterium that resides in the mucus layer, but its potential in intestinal inflammatory diseases has sparked controversy. It is well known that both the consumption of fructose-containing beverages and psychological stress increase the risk of intestinal disease. Our results revealed that a high-fructose diet aggravated the damage to the jejunal mucosal barrier caused by restraint stress, reduced tight junction protein expression and the intestinal digestion and absorption capacity, disrupted the ability of Paneth cells to secrete antimicrobial peptides, and promoted the expression of inflammatory cytokines. *A. muciniphila* colonization enhanced the defense function of the mucosal barrier by enhancing the function of the NLRP6, promoting autophagy, maintaining the normal secretion of antimicrobial peptides in Paneth cells, promoting the expression of tight junction proteins, negatively regulating the NF-kB signaling pathway and inhibiting the expression of inflammatory cytokines. Our work indicates that *A. muciniphila* ameliorates the disruption of the intestinal mucosal barrier under high fructose and restraint stress. These results provided a rationale for the development of probiotic colonization for the prevention or treatment of intestinal diseases.

## 1. Introduction

It is reported that psychological stress is linked to various gastrointestinal diseases [1,2], damage to the intestinal defense system [3], and induces alterations in the gut microbial community [4]. Intestinal bacterial imbalance can cause intestinal inflammation and subsequent Paneth cell dysfunction [5]. Previous studies have shown that antibiotic treatments and probiotics are beneficial to patients with intestinal diseases [6,7], suggesting that intestinal flora may play an important role in the pathogenesis of intestinal diseases.

*A. muciniphila*, a mucus-degrading bacterium, inhabiting the mucus layer, is a next-generation probiotic with important application prospects [8], and plays a crucial role in regulating the gut barrier and other homeostasis and metabolic functions [9]. It was found that daily administration of live *A. muciniphila* could counteract the development of intestinal barrier dysfunction induced by a high-fat diet [10]. Moreover, research confirmed that administration of *A. muciniphila* ameliorates dextran sulfate sodium-induced ulcerative colitis in mice by enhancing gut barrier function [11,12]. Coupled with this is a growing concern regarding the effect of *A. muciniphila* on intestinal diseases, thus underscoring the need to understand the implications of *A. muciniphila* on the intestinal mucosal barrier. However, the exact physiological mechanism of this bacterium’s influence on intestinal inflammation and regulation of intestinal permeability remains unclear.

The intestinal mucus barrier is the interface between intestinal microbes and host tissue [13]. The mucus layer and host-generated immune factors enhance this barrier [14]. Recent data suggest that the intestinal mucosa maintains the intestinal barrier by secreting antimicrobial peptides produced by Paneth cells for innate immunity [15,16]. However, Paneth cell dysfunction contributes to intestinal mucosal dysbiosis, as demonstrated in both patient and animal models of inflammatory bowel disease [17,18,19]. 

Notably, accumulating evidence demonstrates that environmental factors and dietary preferences [20,21] could also trigger Paneth cell dysfunction. Some dietary components, such as fructose, can alter the intestinal microbiota and disrupt the integrity of the intestinal epithelial barrier in mice [22]. As high-fructose diets become more common in the Western world, understanding the effects of fructose on human health is critical. Although fructose contributes to many metabolic disorders [23], until now, our understanding of its effects on the intestinal diseases has been lacking.

We hypothesized that restraint stress and a high fructose diet are a potent trigger for gut mucosal barrier disruption and Paneth cell dysfunction. Herein, we showed that high fructose aggravated the damage to the intestinal mucosal barrier caused by restraint stress, reduced the intestinal digestion and absorption capacity, developed Paneth cell defects, inhibited the expression of antimicrobial peptides, increased intestinal apoptosis, and disrupted inflammasome 6 function and intestinal autophagy levels, while *A. muciniphila* colonization alleviated the deterioration of the intestinal barrier caused by these stimuli. Moreover, *A. muciniphila* colonization enhanced the defense function of the mucosal barrier by enhancing the function of the NLRP6, promoting autophagy, maintaining the normal secretion of antimicrobial peptides in Paneth cells, promoting the expression of tight junction proteins, negatively regulating the NF-KB signaling pathway, and inhibiting the expression of inflammatory cytokines. This provided an important theoretical basis for *A. muciniphila* to be developed into the next generation of probiotics that contribute to selective microbiota transplantation, and implies that environmental factors and dietary preferences may trigger the development of small intestinal inflammatory bowel disease.

## 2. Materials and Methods

### 2.1. Animals

Eighty female C57BL/6J mice aged 5-6 weeks, weighing 17–19 g, were purchased from Vital River Laboratory Animal Technology Co., Ltd. (Beijing, China). All animals were grouped under specific pathogen free (SPF) conditions (5 animals per cage), at 22 °C, 12 h of light and 12 h of darkness, and free diet and water. Before the experiment began, the mice were acclimated to the laboratory environment for 7 day. The experimental protocols were approved by the Ethics Committee for Animal Experiments of the China Agricultural University, under permit no. AW11011202-2-1 (Beijing, China).

Experiment I: Restraint stress and high fructose feeding

**Figure 1:**
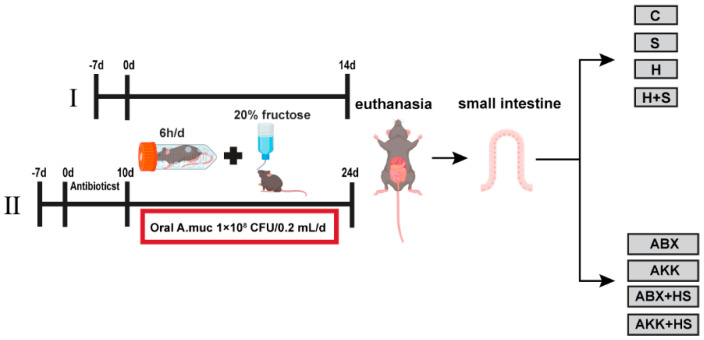
Study design of the animal experiment.

The experimental design has been shown in Figure 1. Forty mice were randomly assigned to four groups: control group (C; *n* = 10), restraint stress group (S; *n* = 10), high fructose group (H; *n* = 10), and high fructose + restraint stress group (H + S; *n* = 10). The restraint stress procedure follows the approach previously reported [24,25]. Briefly, mice in the S and H + S groups were confined to a 50 mL well-ventilated centrifuge tube for 6 h a day (10:00 a.m. to 16:00 a.m.) for 14 consecutive days, during which time they were not allowed to move back and forth during the operation. Then, 20% fructose was added to the drinking water of the mice in the H + S group.

Experiment II: Antibiotic treatment and colonization with *A. muciniphila* bacteria

The experimental design has been shown in Figure 1. Forty mice were randomly assigned to four groups: antibiotic treatment group (ABX; *n* = 10), *A. muciniphila* colonization group (AKK; *n* = 10), antibiotic treatment + high fructose and restraint stress group (ABX + HS; *n* = 10), and *A. muciniphila* colonization + high fructose and restraint stress group (AKK + HS; *n* = 10). Antibiotics were added to the drinking water of the above four groups of mice for treatment for 10 consecutive days: for antibiotic treatment, 1 g/L ampicillin, 100 mg/L gentamicin, 0.5 g/L neomycin, 0.5 g/L vancomycin, and 10 mg/L erythromycin, with continuous drinking water for 10 days of antibiotic treatment. All antibiotics were obtained from Solarbio Science & Technology Co., Ltd. (Beijing, China). After the antibiotic treatment, the mice in the AKK and AKK + HS groups were treated with *A. muciniphila* by oral gavage at a dose of 1 × 10^8^ CFU/0.2 mL, every day for 14 consecutive days. The control group was orally administered an equal volume of sterile anaerobic PBS. High fructose and restraint stress treatments were the same as experiment I.

At the end of the treatment, a total of 80 mice in the two groups of animal experiments were euthanized for cervical dislocation and analyzed. Fecal samples of all mice were collected sterilely and stored at −80 °C for future analysis. The 4–5 mm segments of small intestine were rinsed with PBS and then fixed with 10% formalin for subsequent histological analysis, and the rest of the small intestine tissue was rinsed with PBS and frozen in liquid nitrogen for subsequent analysis.

### 2.2. Histological Analysis 

The small intestine fixed in 10% formalin was embedded in paraffin. Specimens were subsequently cut to 5 μm thickness and stained with hematoxylin and eosin (H&E) and periodic acid–Schiff and Alcian blue (PAS/AB). Villus height (V), crypt depth (C), and the V/C ratio were measured using ImageJ software. Dark blue staining indicates goblet cells. The number of goblet cells was counted by Image J software and expressed as positive cells per villus.

### 2.3. Immunohistochemistry

After deparaffinization and rehydration, sections were soaked in sodium citrate buffer for heat-induced epitope exposure. Nonspecific binding sites were blocked by incubating with 10% goat serum at 37 °C for 1 h. Then, sections were incubated with anti–Ki-67 antibody (1:200; ab15580, Abcam, Cambridge, CA, USA) overnight at 4 °C, followed by incubation with biotinylated goat anti-rabbit IgG secondary antibodies (1:300, A0277, beyotime Co., Ltd., Shanghai, China) for 2 h. Following the washing, the sections were incubated sequentially with 1:300 HRP-streptavidin (1:300, A0303, beyotime Co., Ltd., Shanghai, China) for 2 h. After, they were treated with diaminobenzidine (DAB) Kit (PV-6001, ZSGB Biotech, Inc., Beijing, China) and counterstained with hematoxylin. The average integrated optical density (IOD) of the positive cells was measured by ImageJ software.

### 2.4. RNA Isolation and RT–qPCR Analysis

Total RNA from jejunum tissue was extracted by using TRIzol (Invitrogen). RNA was reverse-transcribed using the HiScript III 1st Strand cDNA Synthesis Kit (+gDNA wiper) (Vazyme Biotech Co., Ltd., Naijing, China) according to the manufacturer’s instructions. The relative mRNA expression levels were determined with SYBR green master mix (Q141-02; Vazyme Biotech Co., Ltd., Naijing, China) by qPCR using the Step One Plus Real-Time PCR system (Applied Biosystems, Waltham, MA, USA). See Supplementary Table S1 for a list of primer sequences used for RT-qPCR.

### 2.5. Terminal Deoxynucleotidyl Transferase-Mediated dUTP Nick End Labeling (TUNEL) Assay

The formalin-fixed jejunum sections were subjected to TUNEL staining using a TUNEL assay kit (T2190, Solarbio, China). Nuclei were stained and images were acquired using a fluorescent microscope (Nikon Instruments, Inc., Melville, NY, USA). We selected at least 6 fields of view, and the number of TUNEL+ cells was counted using ImageJ (National Institutes of Health, Bethesda, MD, USA).

### 2.6. Isolation and Cultivation of A. muciniphila Strains

*A. muciniphila* (ATCC-BAA-835), purchased from Testtop Biotechnology Co., Ltd. (Ningbo, China), was labeled on brain heart perfusion (BHI) agar supplemented with 0.5% porcine mucin and 0.05% cysteine under anaerobic conditions. After 48 h of incubation at 37 °C in an anaerobic jar, bacteria were harvested from the plates, suspended in glycerol-containing anaerobic phosphate-buffered saline (PBS), then dispensed and stored at −80 °C. *A. muciniphila* suspended in 200 μL anaerobic PBS (1.0 × 10^8^ colony-forming units per mouse) was continuously gavaged to mice for 14 d.

### 2.7. Western Blotting

Frozen jejunum samples were homogenized with RIPA lysis buffer (P0013B, Beyotime Biotechnology, Shanghai, China) to extract total proteins. The homogenate was centrifuged at 12,000× *g* at 4 °C for 20 min. Protein content was quantified using a bicinchoninic acid (BCA) protein detection kit (CW0014S; CoWin Biotech Co., Inc., Beijing, China) according to the manufacturer’s instructions. The protein samples were boiled at 99 °C for 10 min, electrophoresed on a 10–15% SDS polyacrylamide gel, and transferred to a PVDF membrane (Millipore, Billerica, MA, USA). The samples were then blocked with 5% nonfat milk in Tris-buffered saline solution Tween 0.1% for 1.5 h at room temperature. Subsequently, membranes were incubated with rabbit antibodies against Bcl-2 antibody (1:1000, 12789-1-AP, Proteintech, Wuhan, China), cleaved-Caspase-3 antibody (1:1000, 9661, Santa Cruz, Dallas, TX, USA), cleaved-PARP antibody (1:1000, CSB-PA000080, Wuhan, China), ATG7 (1:1000, CSB-PA002294LA01HU, Wuhan, China), ATG5-ATG12 (1:1000, A0731, Sigma, MO, USA), LC3 (1:1000, NB100-2220, Novus, Beijing, China), Claudin-1 antibody (1:3000, ab15098, Abcam, Cambridge, CA, USA), Claudin-3 antibody (1:1000, ab15102, Abcam, Cambridge, CA, USA), ZO-1 antibody (1:1000, ab276131, Abcam, Cambridge, CA, USA), Occludin antibody (1:1000, ab216327, Abcam, Cambridge, CA, USA), Phospho-IkB (1:1000, ab133462, Abcam, Cambridge, CA, USA), Phospho-NF-kB p65 (1:1000, ab76302, Abcam, Cambridge, CA, USA), NLRP6 (1:1000, 144-61128-50, Raybiotech, Wuhan, China), Caspase-1 (1:1000, 22915-1-AP, Proteintech, Wuhan, China), ASC/TMS1 (1:1000, 67824S, Cell Signaling Technology, Boston, MA, USA), mouse antibodies against SQSTM1/p62 antibody (1:1000, 88588, Santa Cruz, Dallas, TX, USA), and anti-β-actin antibody (1:10,000; 66009-1-lg, Proteintech, Wuhan, China), overnight at 4 °C. Blots were washed and incubated with the HRP-conjugated goat anti-mouse IgG (1:10,000, SA00001-1, Proteintech, Wuhan, China) (for β-actin) or goat anti-rabbit IgG (1:10,000, SA00001-2, Proteintech, Wuhan, China) for 1.5 h at room temperature, and then developed with enhanced chemiluminescence (WBKLS0500, Millipore, Billerica, MA, USA). Densities of protein bands were quantified using ImageJ software (National Institutes of Health, Bethesda, MD, USA).

### 2.8. Immunofluorescence Staining

At least four consecutive sections of the same jejunum tissue were selected; after deparaffinization of jejunum sections, antigen retrieval was performed with 10 mM pH 6.0 sodium citrate solution in a 95 °C water bath for 10 min, followed by a 20 min incubation at room temperature. Slides were washed, blocked in 5% normal goat serum at 37 °C, and stained using the primary antibodies rabbit anti-lysozyme antibody (1:200, ab108508, Abcam, Cambridge, CA, USA) and rabbit anti-NLRP6 antibody (1:1000, 144-61128-50, Raybiotech, Shanghai, China) overnight at 4 °C, respectively. Then, rabbit anti–lysozyme antibody-stained sections were incubated with goat anti-rabbit Alexa Fluor 594 (1:300, ab150080, Abcam, Cambridge, CA, USA), and rabbit anti-NLRP6 antibody-stained sections were incubated with goat anti-rabbit Alexa Fluor 488 (1:400, ab150077, Abcam, Cambridge, CA, USA). The sections were photographed with a Nikon Eclipse TE 2000S inverted microscope (Nikon Instruments Co., Inc., New York, NY, USA). The same position of the serial sections stained with the two antibodies were photographed and counted, respectively. The numbers of positively stained puncta were counted using Image-Pro Plus software (Media Cybernetics, Rockville, MD, USA). 

### 2.9. Statistical Analysis

Data were presented either as mean ± standard error of mean (SEM). Data analysis was performed by GraphPad Prism 8.0 program (GraphPad Software Co., Inc., San Diego, CA, USA). One-way ANOVA and Tukey’s multiple comparison test or two-way ANOVA with Tukey’s post hoc test were used to compare multiple groups. *p* < 0.05 was considered statistically significant.

## 3. Results

### 3.1. Colonization of A. muciniphila Alleviated Intestinal Mucosal Barrier Disruption by High Fructose and Restraint Stress

**Figure 2:**
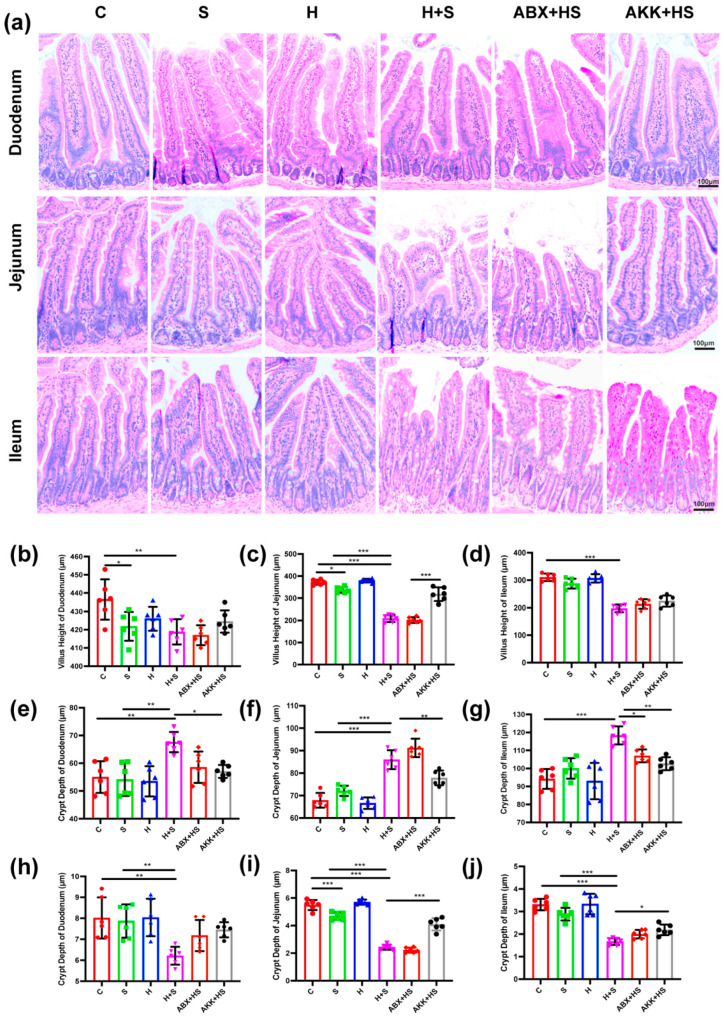
Changes of intestinal morphology after *A. muciniphila* colonization in mice stimulated by high fructose and restraint stress. (**a**) H.E. staining in duodenum, jejunum and ileum of C, S, H, H + S, ABX + HS and AKK + HS groups, respectively (scale: 100 μm) (*n* = 6). Villus height (**b**–**d**), crypt depth (**e**–**g**) and V/C ratio (**h**–**j**) were measured in duodenum (**b**,**e**,**h**), jejunum (**c**,**f**,**i**) and ileum (**d**,**g**,**j**) of C (red), S (green), H (blue), H + S (rose red), ABX + HS (tangerine) and AKK + HS groups (black), respectively. Data are presented as the mean ± SEM. * *p* < 0.05, ** *p* < 0.01 and *** *p* < 0.001 indicate significant difference. C: control group; S: restraint stress; H: high fructose; H + S: high fructose and restraint stress; ABX + HS: antibiotic treatment + high fructose and restraint stress group; AKK + HS: *A. muciniphila* colonization + high fructose and restraint stress group.

H.E. staining results showed that duodenum, jejunum and ileum tissues were damaged and deformed to varying degrees in the H + S group. The villus lengths of duodenum (*p* = 0.004), jejunum (*p* < 0.001) and ileum (*p* = 0.002) were significantly decreased in the H + S group, compared with the C group, respectively (Figure 2a–d). Meanwhile, the crypt depth of the duodenum (*p* = 0.003), jejunum (*p* < 0.001) and ileum (*p* = 0.011) were significantly increased in H + S group (Figure 2e–g), which resulted in a notable decrease in the V/C, compared with the C group. In terms of V/C ratio, the jejunum (*p* < 0.001) was the most seriously injured compared with that of the C group, followed by the duodenum (*p* = 0.005) and the ileum (*p* = 0.002) (Figure 2h–j). These results suggested that both high fructose and restraint stress had different degrees of damage to small intestinal segments. Moreover, the addition of high fructose could aggravate the injury of intestinal morphology. However, colonization of *A. muciniphila* alleviated the damage to the jejunum in the AKK + HS group compared with the H + S group; in particular, the villus height (*p* < 0.001) and V/C ratio (*p* < 0.001) significantly increased, and crypt depth significantly decreased (*p* = 0.004) (Figure 2c,f,i).

**Figure 3:**
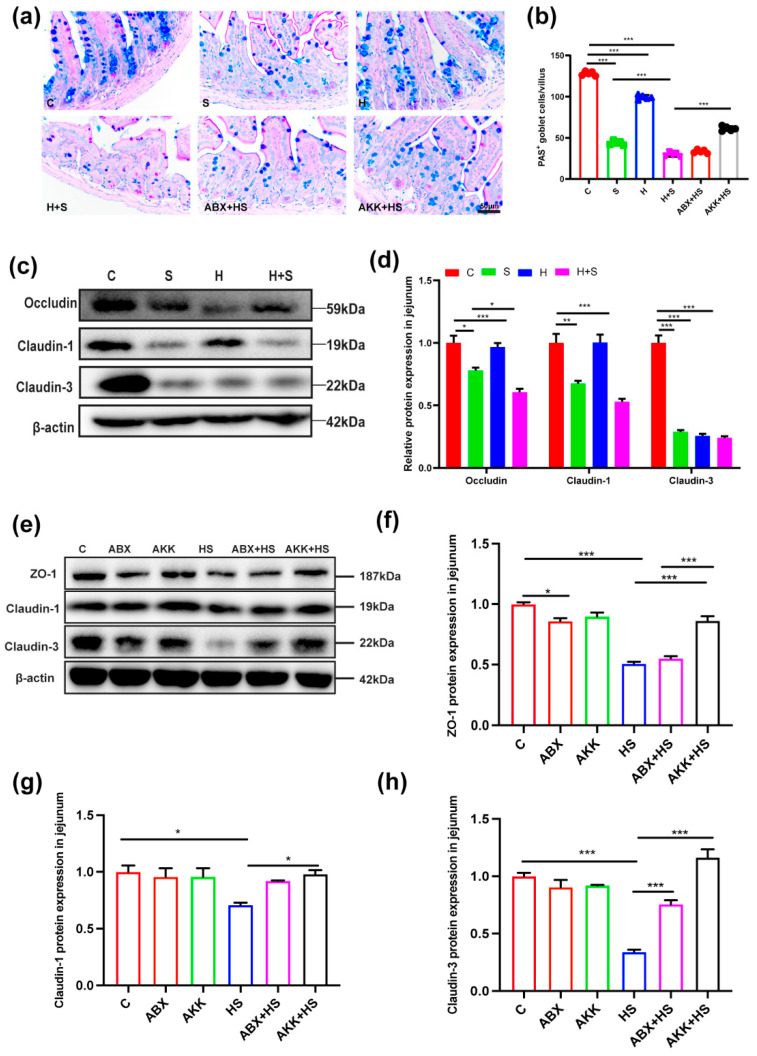
Changes of goblet cells and tight junction protein after *A. muciniphila* colonization in mice stimulated by high fructose and restraint stress. (**a**) AB-PAS staining (scale: 50 μm) in jejunum of C, S, H, H + S, ABX + HS and AKK + HS groups, respectively (*n* = 5). (**b**) The number of goblet cells expressed as positive cells per villus in jejunum of C (red), S (green), H (blue), H + S (rose red), ABX + HS (tangerine) and AKK + HS groups (black), respectively. (**c**) The expression of jejunum tight junction protein occludin, claudin-1, claudin-3 and β-actin protein were examined by Western blotting in the C, S, H, H + S groups. (**d**) The relative protein levels were normalized to β-actin (*n* = 5). (**e**) The expression of the jejunum tight junction protein ZO-1, claudin-1, claudin-3 and β-actin protein were examined by Western blotting. (**f**–**h**) The relative protein levels were normalized to β-actin in the C (red), ABX (tangerine), AKK (green), H + S (blue), ABX + HS (rose red) and AKK + HS (black) groups (*n* = 5). Data are presented as the mean ± SEM. * *p* < 0.05, ** *p* < 0.01 and *** *p* < 0.001 indicate significant difference. C: control group; S: restraint stress; H: high fructose; H + S: high fructose and restraint stress; ABX: antibiotic treatment group; AKK: *A. muciniphila* colonization group; ABX + HS: antibiotic treatment + high fructose and restraint stress group; AKK + HS: *A. muciniphila* colonization + high fructose and restraint stress group.

Since the destruction of the jejunum by high fructose and restraint stress was most pronounced, we focused on the changes in the jejunum. Firstly, to assess the effect on the jejunal mucosal barrier, mucin-secreting goblet cells were measured using AB-PAS staining; this staining presented goblet cells in blue, and indicated that they were mainly distributed in the lower half of intestinal villi. The number of goblet cells in the jejunum were also significantly reduced in the H + S group (*p* < 0.001) compared to the control group (Figure 3a,b). However, after *A. muciniphila* colonization, the number of goblet cells in the jejunum was significantly increased in the AKK + HS group (*p* < 0.001) compared with the H + S group (Figure 3a,b).

Then we detected the expression of jejunal tight junction proteins by Western blotting. The expression of tight junction proteins occludin (*p* = 0.000), claudin-1 (*p* = 0.001), and claudin-3 (*p* < 0.001) were significantly decreased in the H + S group, compared with the C group (Figure 3c,d). In contrast, after *A. muciniphila* colonization, the expression of ZO-1 (*p* < 0.001), claudin-1 (*p* = 0.028) and claudin-3 (*p* < 0.001) were significantly increased in the AKK + HS group compared with the H + S group (Figure 3e–h). In addition, we used a combination of antibiotics administered before the high fructose and restraint stress, and the expression of claudin-3 (*p* < 0.001) was significantly higher in the ABX + HS group than in the H + S group (Figure 3e,h), indicating that the presence of microbiota play a critical effect on the expression of tight junction proteins.

### 3.2. Colonization of A. muciniphila Prevented the Decrease of Paneth Cell Number and Improved the Expression of Antimicrobial Peptides by High Fructose and Restraint Stress

We next investigated whether the distribution of Paneth cells changed upon high fructose and restraint stress in mice. Paneth cells play a role in intestinal defense and self-renewal [26]. Firstly, the Paneth cells marker, lysozyme (LZM), in the jejunum was measured by immunofluorescence. It was found to be significantly reduced in the H + S group (*p* < 0.001), compared with the C group (Supplementary Figure S1a,b). In contrast, in the AKK + HS group (*p* < 0.001), the number of Paneth cells was significantly higher than that in the H + S (Supplementary Figure S1a,b).

The expression of antimicrobial peptide genes Retnlb (*p* = 0.001), Itln1 (*p* = 0.023), Ang4 (*p* = 0.001) Lyz1 (*p* < 0.001), Lyz2 (*p* = 0.000), Cryptdins (*p* = 0.002), Muc2 (*p* = 0.004), Defcr1 (*p* = 0.000), Defcr2 (*p* < 0.001), Defcr4 (*p* = 0.002) and Defa6 (*p* = 0.000) were also significantly decreased in the H + S group compared with the control group (Supplementary Figure S2a–c). However, *A. muciniphila* colonization improved antimicrobial peptide Defcr1 (*p* = 0.001), Lyz1 (*p* = 0.004), Cryptdins (*p* = 0.019) and Retnlb (*p* = 0.000) expression compared with the H + S group (Supplementary Figure S2e). Together, these data suggest that high fructose and restraint stress inhibits the expression of antimicrobial peptides, but can be improved by *A. muciniphila* colonization.

### 3.3. Colonization of A. muciniphila Inhibited the Expression of Inflammatory Cytokines, and Activation of NF-κB Pathways in High-Fructose-Stressed Mice

**Figure 4:**
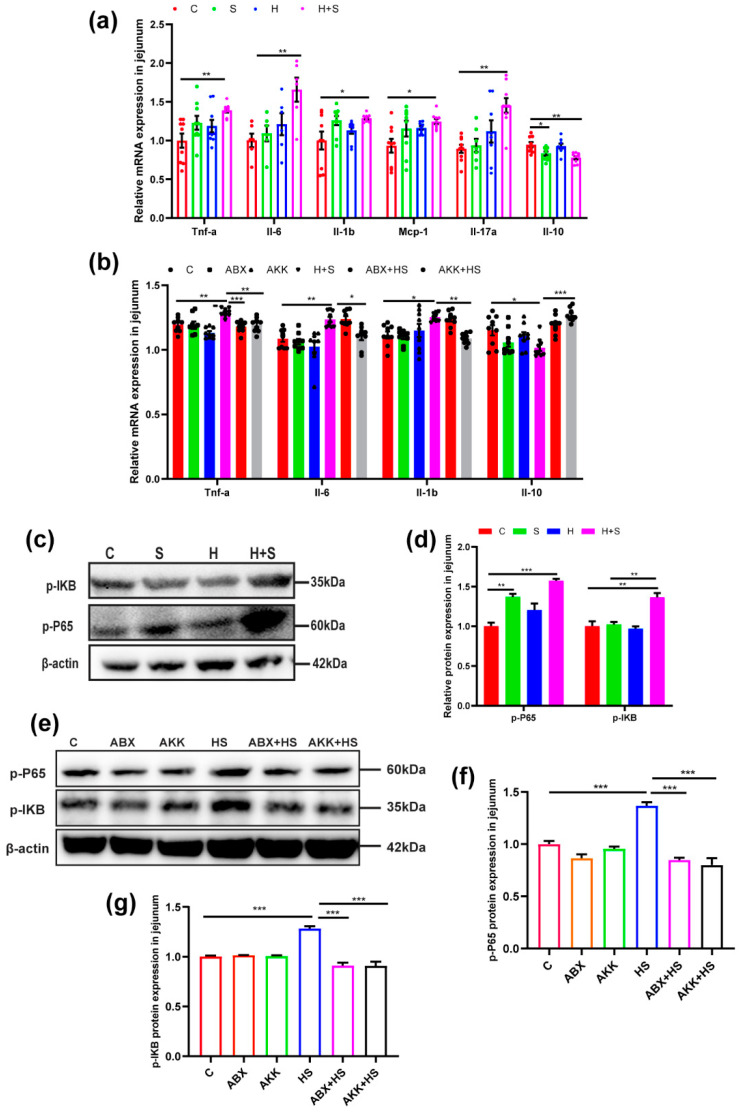
Effects of *A. muciniphila* colonization on the mRNA levels of inflammatory cytokines and NF-kB signaling pathway protein expression in the jejunum of mice under high fructose and restraint stress. (**a**) The expression of mRNA levels of inflammatory cytokine Tnf-a, Il-6, Il-1b, Mcp-1, Il-17a and Il-10 in the C, S, H and H + S groups. (*n* = 9). (**b**) Expression of mRNA levels of inflammatory cytokine Tnf-a, Il-6, Il-1b and Il-10 in the C, ABX, AKK, H + S, ABX + HS and AKK + HS groups. (*n* = 9). (**c**) The expression levels of NF-kB signaling pathway proteins p-P65, p-IkB and β-actin were examined by Western blotting in the C, S, H, H + S groups. (**d**) The relative protein levels were normalized to β-actin (*n* = 5). (**e**) The expression of NF-kB signaling pathway proteins p-P65, p-IkB and β-actin were examined by Western blotting. (**f**,**g**) The relative protein levels were normalized to β-actin in the C (red), ABX (tangerine), AKK (green), H + S (blue), ABX + HS (rose red) and AKK + HS (black) groups (*n* = 5). Data are presented as the mean ± SEM. * *p* < 0.05, ** *p* < 0.01 and *** *p* < 0.001 indicate significant difference. C: control group; S: restraint stress; H: high fructose; H + S: high fructose and restraint stress; ABX: antibiotic treatment group; AKK: *A. muciniphila* colonization group; ABX + HS: antibiotic treatment + high fructose and restraint stress group; AKK + HS: *A. muciniphila* colonization + high fructose and restraint stress group.

Firstly, mRNA levels of inflammatory cytokines in jejunum tissue were detected. We found that the expression of Tnf-a (*p* = 0.006), Il-6 (*p* = 0.007), Il-1b (*p* = 0.030), Mcp-1 (*p* = 0.019) and Il-17a (*p* = 0.001) were significantly increased, and Il-10 (*p* = 0.002) was significantly reduced in the H + S group, compared with the C group (Figure 4a). To further evaluate the impact of *A. muciniphila* colonization on intestinal inflammation caused by high fructose and restraint stress, *A. muciniphila* colonization inhibited the expression of Tnf-a (*p* = 0.004), Il-6 (*p* = 0.023), Il-1b (*p* = 0.001) and promoted the expression of Il-10 (*p* < 0.001) in the AKK + HS group compared with the H + S group (Figure 4b).

Through Western blotting detection, it was shown that high fructose and restraint stress stimulation activated the NF-kB pathway in the jejunum. The protein levels of p-P65 (*p* = 0.000) and p-IkB (*p* = 0.002) were increased in the H + S group, compared with the C group, respectively (Figure 4c,d). After colonization with *A. muciniphila* to H + S mice, however, the protein levels of p-P65 (*p* < 0.001) and p-IkB (*p* < 0.001) were decreased compared to the H + S group. Similarly, the same changes occurred in the ABX + HS group (Figure 4e–g). This suggested that the presence of intestinal flora was critical for NF-kB pathway activation.

### 3.4. Colonization of A. muciniphila Reduced Intestinal Apoptosis and Increased Autophagy Levels by High Fructose and Restraint Stress

**Figure 5:**
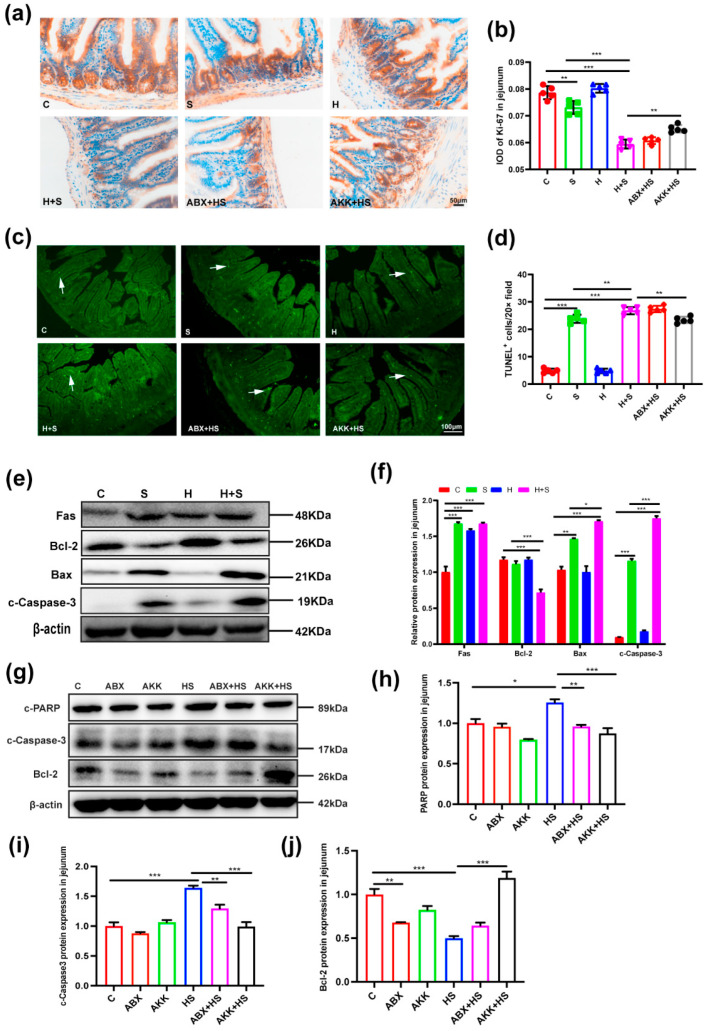
Effects of *A. muciniphila* colonization on jejunal cell proliferation and apoptotic protein expression in mice stimulated with high fructose and restraint stress. (**a**) The immunohistochemical staining of Ki-67 in jejunum sections (scale bar = 50 μm). (**b**) IOD of Ki-67-positive cells in the jejunum of C (red), S (green), H (blue), H + S (rose red), ABX + HS (tangerine) and AKK + HS groups (black), respectively (*n* = 5). (**c**) Apoptosis was measured by TUNEL staining (scale bar = 100 μm). The white arrow shows TUNEL-positive cells (green). (**d**) The number of TUNEL-positive cells per 20 magnification were counted in the jejunum of C (red), S (green), H (blue), H + S (rose red), ABX + HS (tangerine) and AKK + HS groups (black), respectively (*n* = 5). (**e**) Anti-apoptotic protein Bcl-2, apoptotic protein Fas, Bax and c-Caspase-3 production was examined in the C, S, H, H + S groups by Western blotting. (**f**) The relative protein levels were normalized to β-actin (*n* = 5). (**g**) Anti-apoptotic protein Bcl-2, apoptotic protein c-PARP and c-Caspase-3 production were examined in the C, ABX, AKK, H + S, ABX + HS and AKK + HS groups by Western blotting. (**h**–**j**) The relative protein levels were normalized to β-actin in the C (red), ABX (tangerine), AKK (green), H + S (blue), ABX + HS (rose red) and AKK + HS (black) (*n* = 5). Data are presented as the mean ± SEM. * *p* < 0.05, ** *p* < 0.01 and *** *p* < 0.001 indicate significant difference. C: control group; S: restraint stress; H: high fructose; H + S: high fructose and restraint stress; ABX: antibiotic treatment group; AKK: *A. muciniphila* colonization group; ABX + HS: antibiotic treatment + high fructose and restraint stress group; AKK + HS: *A. muciniphila* colonization + high fructose and restraint stress group.

We next validated the impact of high fructose and restraint stress on the levels of apoptosis and autophagy in the jejunum. Firstly, the results of Ki-67 immunohistochemical staining showed that the proliferation ability of jejunal cells in the H + S group (*p* < 0.001) was significantly decreased, compared with the C group (Figure 5a,b). On the contrary, the results of TUNEL fluorescence staining showed that the apoptosis level of jejunal cells in the H + S group (*p* < 0.001) was significantly increased, compared with the C group (Figure 5c,d). The expression levels of apoptotic proteins Fas (*p* < 0.001), Bax (*p* < 0.001) and c-Caspase-3 (*p* < 0.001) were up-regulated in the H + S group, while the expression of anti-apoptotic protein Bcl-2 (*p* = 0.000) was decreased, compared with the C group (Figure 5e,f). However, after colonization with *A. muciniphila* to H + S mice, the number of Ki-67 positive cells (*p* = 0.001) was significantly increased, while the number of TUNEL positive cells (*p* = 0.002) was significantly decreased compared with the H + S group. Moreover, c-PARP (*p* = 0.009) and c-Caspase-3 (*p* < 0.001) were down-regulated, but Bcl-2 (*p* < 0.001) was up-regulated compared with the H + S group (Figure 5g–j).

**Figure 6:**
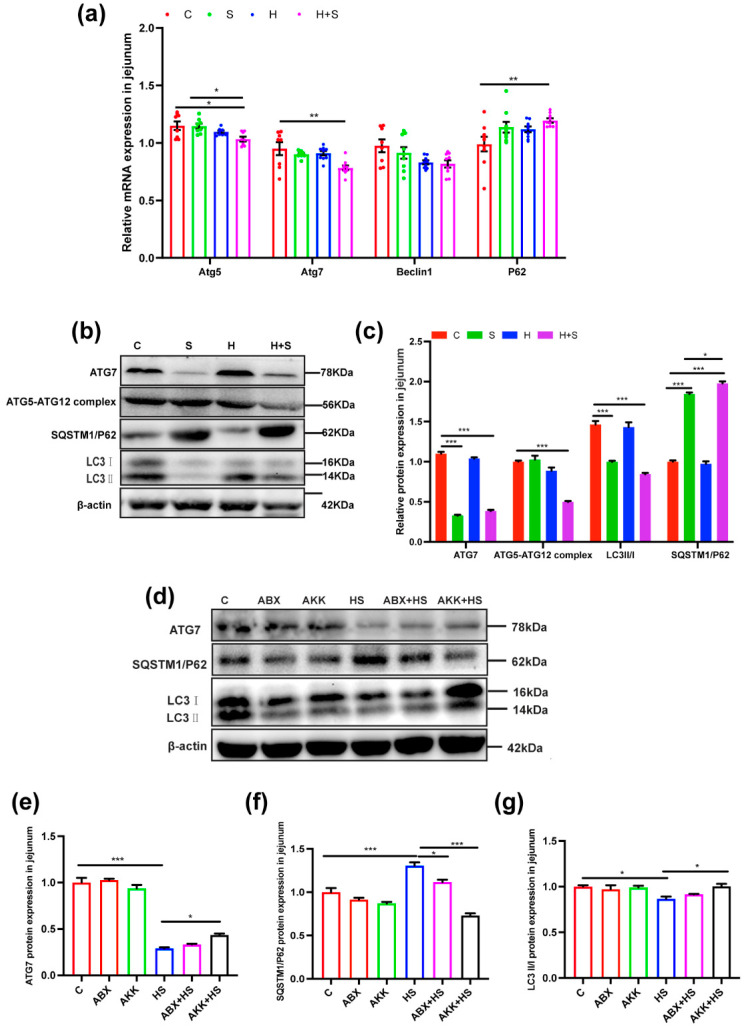
Effects of *A. muciniphila* colonization on jejunal autophagy-related proteins expression in mice stimulated with high fructose and restraint stress. (**a**) Changes in mRNA levels of autophagy-related proteins by real-time qPCR in mice (*n* = 9). (**b**) The expression levels of autophagy-related proteins ATG7, ATG5–ATG12 complex, SQSTM1/P62 and LC3II/I were examined in the C, S, H, H + S groups by Western blotting. (**c**) The relative protein levels were normalized to β-actin (*n* = 5). (**d**) The expression of autophagy-related proteins ATG7, SQSTM1/P62 and LC3II/I were examined in the C, ABX, AKK, H + S, ABX + HS and AKK + HS groups by Western blotting. (**e**–**g**) The relative protein levels were normalized to β-actin in the C (red), ABX (tangerine), AKK (green), H + S (blue), ABX + HS (rose red) and AKK + HS (black) (*n* = 5). Data are presented as the mean ± SEM. * *p* < 0.05, ** *p* < 0.01 and *** *p* < 0.001 compared indicate significant difference. C: control group; S: restraint stress; H: high fructose; H + S: high fructose and restraint stress; ABX: antibiotic treatment group; AKK: *A. muciniphila* colonization group; ABX + HS: antibiotic treatment + high fructose and restraint stress group; AKK + HS: *A. muciniphila* colonization + high fructose and restraint stress group.

Next, we analyzed the changes of mRNA level related to autophagy. The expression level of autophagy gene Atg5 (*p* = 0.012) and Atg7 (*p* = 0.004) were significantly reduced, while P62 (*p* = 0.009) was increased in the H + S group, compared with the C group (Figure 6a). Autophagy protein level analysis further revealed that ATG7 (*p* < 0.001), the ATG5–ATG12 complex (*p* < 0.001) and LC3II/I (*p* = 0.000) were significantly down-regulated, while SQSTM1/P62 (*p* < 0.001) was significantly up-regulated in the H + S group, compared with the C group (Figure 6b,c). However, after colonization with *A. muciniphila* to H + S mice, ATG7 (*p* = 0.013) and LC3II/I (*p* = 0.020) were significantly up-regulated, while SQSTM1/P62 (*p* < 0.001) was significantly down-regulated in the AKK + HS group compared to the H + S group (Figure 6d–g). These results collectively indicated that *A. muciniphila* alleviated the increase of jejunal apoptosis and the decrease of autophagy caused by high fructose and restraint stress. 

### 3.5. Colonization of A. muciniphila Prevented the Destruction of Inflammasome 6 by High Fructose and Restraint Stress

**Figure 7:**
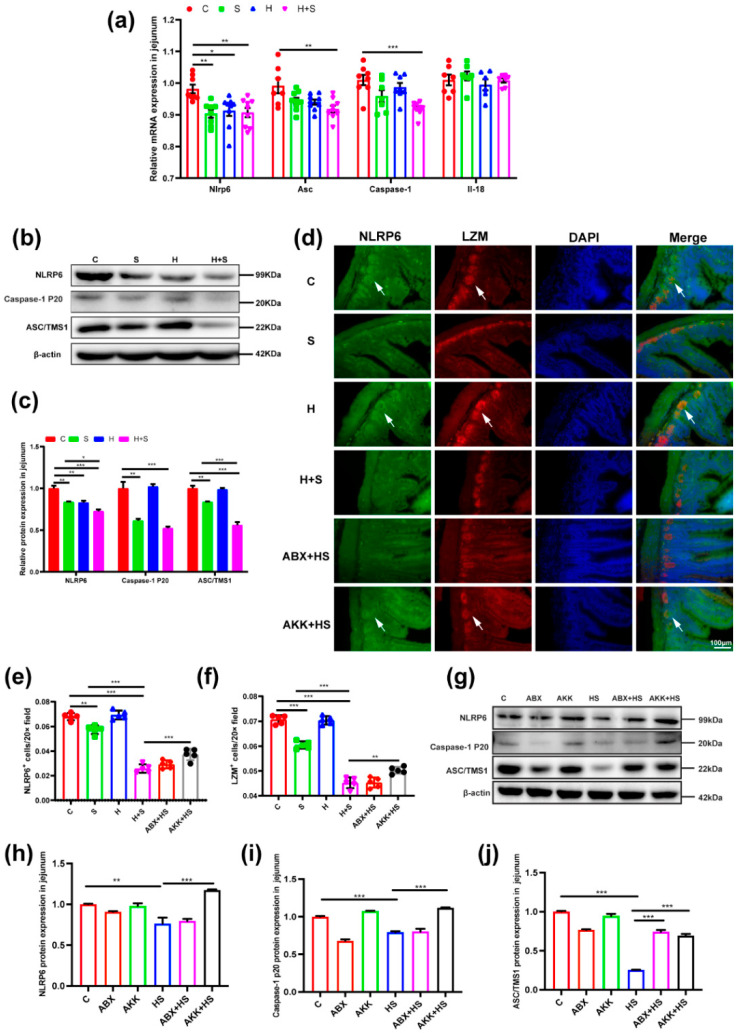
Effects of *A. muciniphila* colonization on the expression of jejunal inflammasome 6 expression in mice stimulated with high fructose and restraint stress. (**a**) Changes in mRNA levels of Nlrp6, Asc, Caspase-1 and Il-18 by real-time qPCR in mice (*n* = 9). (**b**) The expression levels of NLRP6-related proteins were examined in the C, S, H, H + S groups by Western blotting. (**c**) The relative protein levels were normalized to β-actin (*n* = 5). (**d**) Representative immunofluorescence image of the jejunum showed a significant reduction of Paneth cells and NLRP6 levels in the H + S group. Formation of the Paneth cell is visualized utilizing the rabbit anti-LZM antibody expressed protein (red) and rabbit anti-NLRP6 antibody expression protein (green); epithelial cell nuclei are indicated with DAPI (blue). The white arrow represents the region of positive expression. The scale bar represents 50 μm. (**e**) Quantitation of Paneth cell formation through LZM-positive cells (red) in the intestinal crypt of C (red), S (green), H (blue), H + S (rose red), ABX + HS (tangerine) and AKK + HS (black) groups, respectively (*n* = 5). (**f**) Quantification of NLRP6-positive expression (green) in Paneth cells of C (red), S (green), H (blue), H + S (rose red), ABX + HS (tangerine) and AKK + HS groups (black), respectively (*n* = 5). (**g**) The expression of NLRP6-related proteins were examined in the C, ABX, AKK, H + S, ABX + HS and AKK + HS groups by Western blotting. (**h**–**j**) The relative protein levels were normalized to β-actin in the C (red), ABX (tangerine), AKK (green), H + S (blue), ABX + HS (rose red) and AKK + HS (black) (*n* = 5). Data are presented as the mean ± SEM. * *p* < 0.05, ** *p* < 0.01 and *** *p* < 0.001 indicate significant difference. C: control group; S: restraint stress; H: high fructose; H + S: high fructose and restraint stress; ABX: antibiotic treatment group; AKK: *A. muciniphila* colonization group; ABX + HS: antibiotic treatment + high fructose and restraint stress group; AKK + HS: *A. muciniphila* colonization + high fructose and restraint stress group.

Next, we tested whether high fructose and restraint stress stimulation affected the expression of inflammasome 6. The expression of inflammasome 6-related genes were significantly decreased in the H + S group, compared with the C group (Figure 7a). Consistent with mRNA expression level, NLRP6 (*p* < 0.001), Caspase-1 p20 (*p* = 0.000) and ASC/TMS1 (*p* < 0.001) protein expression levels were significantly down-regulated in the H + S group, compared with the C group (Figure 7b,c). However, after *A. muciniphila* colonization, NLRP6 (*p* < 0.001), Caspase-1 p20 (*p* < 0.001) and ASC/TMS1 (*p* < 0.001) expression level were significantly increased in the AKK + HS group, compared to the H + S group (Figure 7g–j).

Moreover, we further verified the expression changes of NLRP6 on Paneth cells. Immunofluorescence staining was performed using LZM and NLRP6, respectively. The results showed that NLRP6 was expressed on Paneth cells, and the trend of NLRP6 expression was consistent with that of Paneth cells. Especially in the H + S (*p* < 0.001) and ABX + HS (*p* < 0.001) groups, it was obvious that the expression level of NLRP6 in the jejunum was significantly decreased, accompanied by a significant decrease in the number of Paneth cells compared with the C group (Figure 7d–f). However, in the AKK + HS group, both the expression of NLRP6 (*p* = 0.001) and the number of Paneth cells (*p* = 0.002) rebounded significantly compared with the H + S group (Figure 7d–f). This indicated that NLRP6 could be expressed on Paneth cells, and the colonization of *A. muciniphila* restored the expression of NLRP6 and the number of Paneth cells.

## 4. Discussion

Environmental factors [27], dietary preferences [28] and intestinal flora [29] are all related to the occurrence and development of intestinal diseases. However, most of the research so far has focused on the destruction of the colon, ignoring studies on the small intestine. Since the small intestine is the body’s main location for digestion and absorption of nutrients, we sought to investigate the effects of restraint stress stimuli and high fructose diet on the mucosal barrier of the small intestine, as well as the regulatory mechanisms involved in the disruption of these stimuli following *A. muciniphila* colonization.

In the current study, first of all, we found that high fructose exacerbated the morphological and functional disruption of small intestinal epithelial cells in C57BL/6J mice by restraint stress stimulation. The morphological study showed that villus height, V/C ratio and goblet cell number were significantly decreased in the H + S group. Intestinal villus length, crypt depth and V/C ratio are important indicators to measure the digestion and absorption function of the small intestine [30]. Goblet cells secrete mucin as the first line of defense in the intestinal mucosal barrier [31]. Psychological stress [32] and excessive fructose [33] intake led to deterioration of the intestinal barrier. We found that the expression levels of the tight junction proteins occludin, claudin-1 and claudin-3 were significantly decreased in the jejunum of the H + S group. Moreover, high fructose intake aggravated the reduction of tight junction proteins (occludin, claudin-1) caused by restraint stress stimulation. The level of tight junction protein expression directly affects the integrity and permeability of the intestinal mucosa [34]. Excessive intake of fructose has been found to lead to gut barrier deterioration and endotoxemia [35].

We also evaluated the effect of high fructose and restraint stress on Paneth cells and antimicrobial peptide expression. Paneth cells secrete large amounts of antibacterial and inflammation-related proteins and are major contributors to the intestinal mucosal barrier [15,36]. As expected, high fructose aggravated the inhibition of the number of jejunum Paneth cells and antimicrobial peptides expression stimulated by restraint stress. The above results suggested that high-fructose diet aggravated the damage to the intestinal mucosa, disturbed the intestinal mechanical barrier, and reduced the intestinal digestion and absorption capacity caused by restraint stress, suggesting that our findings have pathophysiological significance. This highlights a potential vulnerability of intestine exposed to fructose when facing stress environments.

A key strength of our study is the assessment of the impact of whether *A. muciniphila* colonization mitigated disruption of the small intestinal mucosal barrier by high fructose and restraint stress. As expected, after colonization of *A. muciniphila*, the reduction of intestinal mucosal barrier disruption caused by high fructose and binding stress was observed. Some striking observations included the increase in the number of goblet cells and Paneth cells, and the promotion of the expression of tight junction proteins and antimicrobial peptides. Previous research also demonstrated that *A. muciniphila* increased the expression of Reg3g [10]. This is consistent with our findings that colonization of *A. muciniphila* in the intestine increases the number of goblet cells and up-regulates the expression of tight junction proteins in the gut [10,37]. A previous study has revealed that *A. muciniphila* protects against psychological disorder-induced colonic mucosal barrier damage and aggravation of colitis [38]. Moreover, studies have found that extracellular vesicles from *A. muciniphila* Muc^T^ (AmEVs) have been found to reduce gut permeability through the regulation of tight junctions in mice [39]. Interestingly, whatever the form of *A. muciniphila* Muc^T^ used (alive or pasteurized, or even AMUC_ 1100 protein), it was proved to strengthen the intestinal barrier [40]. In summary, we provided new experimental evidence for a critical role of colonization of *A. muciniphila* in alleviating the damage of high fructose and restraint stress on the mucosal barrier of the small intestine.

Next, we further investigated the protective mechanism of *A. muciniphila* against the damage of intestinal mucosal barrier by high fructose and restraint stress. We found that high fructose and restraint stress-induced elevated expression levels of pro-inflammatory cytokines, which was caused by activation of the NF-kB signaling pathway, were significantly reversed by *A. muciniphila*. There is some evidence that mice exposed to fructose have increased LPS-induced systemic inflammation [41]. Consistent with our findings, *A. muciniphila* attenuates high-fat diet-induced mRNA levels of pro-inflammatory cytokines IL-6 and IL-1β [42]. Previous studies have found that during the repair of colonic mucosal injury, the enrichment of *A. muciniphila* stimulated proliferation and migration of enterocytes adjacent to the colonic wounds [43]. A recent study showed that *A. muciniphila* exhibits anti-inflammatory properties in the treatment of constipation-predominant irritable bowel syndrome (C-IBS) patients [44].

Coupled with this, we found that *A. muciniphila* colonization inhibited the expression of apoptotic proteins induced by high fructose and restraint stress. Interestingly, studies have found that under the condition of intestinal inflammation, the damage of the intestinal barrier and the increase of the apoptosis level in mice were caused by an autophagy defect [45]. Therefore, we detected the changes of intestinal autophagy level. We were surprised to find that *A. muciniphila* colonization changed the intestinal autophagy reduction level caused by high fructose and restraint stress stimulation. Autophagy is indispensable in regulating cell stress and coordinating host defense response [46]. Additionally, the importance of an intact autophagy system in Paneth cells is strongly supported by clinical observations [47,48]. In previous mice studies, loss of autophagy in Paneth cells led to impaired intestinal permeability [49]. However, the mechanism of Paneth cells regulating autophagy is not completely clear. 

At present, a growing number of studies have found that pyrin-domain containing (NLRP)-6 inflammasome is the nucleotide binding oligomerization domain protein-like receptors, which is essential for maintaining intestinal homeostasis mucosal self-renewal and proliferation [50,51]. Since NLRP6 is highly expressed in intestinal epithelial cells [50,51], it was found that the expression levels of inflammasome 6-related proteins were significantly decreased in the H + S group of the jejunum. Consistent with our results, WAS-induced small bowel inflammation (enteritis) is associated with inhibition of NLRP6 [52], and a high fructose diet results in intestinal epithelial barrier damage and NLRP6 dysfunction [53]. Interestingly, a study claimed that NLRP6 deficiency results in defective goblet cell autophagy and blocks mucus secretion into the colon lumen [54]. Does that have a similar effect on Paneth cells? Our experimental results verified this hypothesis. This suggested that the activity of the NLRP6 inflammasome is critical for autophagy induction and Paneth cells. However, after *A. muciniphila* colonization, the number of Paneth cells in the jejunum increased, and the function of secreting antimicrobial peptides was enhanced, which was synchronized with the increase of NLRP6 expression level. This suggested that *A. muciniphila* may activate inflammasome NLRP6, and then regulate autophagy to affect the function of the Paneth cell, thus participating in mucosal defense.

Finally, antibiotics that removed intestinal flora prevented the partial destruction of the small intestine by high fructose and restraint stress. These results suggest that signals from the gut microbiome are significant contributors to the development of high fructose and restraint stress-induced disruption of the small intestinal mucosal barrier. Another study also found that antibiotics that removed intestinal flora had been shown to significantly reduce alcohol-induced increases in Paneth cell endoplasmic reticulum stress and IL-18 cleavage in the small intestine [55]. In addition, antibiotic depletion of gut microbiota prevents loss of Paneth cells in *T. gondii*-infected mice [49].

In conclusion, we describe the novel finding that beneficial *A. muciniphila* bacteria alleviated the destruction of the small intestine by high fructose and restraint stress stimulation. This was achieved by enhancing the function of inflammasome NLRP6, promoting autophagy, maintaining the normal secretion of antimicrobial peptides in Paneth cells, improving the expression of tight junction proteins, and inhibiting the occurrence of inflammatory reactions, so as to tackle the challenges of intestinal diseases. Our findings indicate that *A. muciniphila* is expected to be a potential probiotic for protection. These results provide a rationale for the development of a treatment with probiotic colonization for the prevention or treatment of intestinal diseases. However, the mechanism by which *A. muciniphila* exerts this protective effect has not been thoroughly studied, and this important research will need to be carried out in the future. Of course, in the future, more in vitro and in vivo studies are needed to further explore the effectiveness and safety of *A. muciniphila* in the treatment of intestinal diseases, and to provide more theoretical support for its development into the next generation of probiotics.

## Data Availability

All relevant data are within the manuscript.

## Supplementary Materials

The following supporting information can be downloaded at: https://www.mdpi.com/article/10.3390/nu14153164/s1, Figure S1: Changes of jejunal Paneth cells after A. muciniphila colonization in mice stimulated by high fructose and restraint stress; Figure S2: Effects of A. muciniphila colonization on the expression of jejunal antimicrobial peptides in high fructose and stress-stimulated mice; Table S1: primers for real-time PCR.
