# Supplementary material for: Akkermansia muciniphila Colonization Alleviating High Fructose and Restraint Stress-Induced Jejunal Mucosal Barrier Disruption
Source: Nutrients. 2022 Jul 30;14(15):3164. doi: 10.3390/nu14153164 (PMC9370786; doi:10.3390/nu14153164)
Supplement: Supplementary file 1 [file nutrients-14-03164-s001.zip › nutrients-1803458-supplementary.pdf]

Supplementary Table S1: primers for real-time PCR

Gene name	Forward	Reverse	source
Cryptdins	GGTGATCATCAGACCCCAGCATCAGT	AAGAGACTAAAACTGAGGAGCAGC	NM_001270613.1
Defcr1	TCAAGAGGCTGCAAAGGAAGAGAAC	TGGTCTCCATGTTCAGCGACAGC	NM_010031.2
Defcr4	CCAGGGGAAGATGACCAGGCTG	TGCAGCGACGATTTCTACAAAGGC	NM_183253.3
Defcr2	CCAGGCTGATCCTATCCAAA	GTCCCATTCATGCGTTCTCT	XM_021155240.1
Defa6	CCTTCCAGGTCCAGGCTGAT	TGAGAAGTGGTCATCAGGCAC	NM_001024225.2
Lyz1	GCCAAGGTCTACAATCGTTGTGAGTTG	CAGTCAGCCAGCTTGACACCACG	NM_013590.4
Lyz2	GGCTGGCTACTATGGAGTCAGCCTG	GCATTCACAGCTCTTGGGGTTTTG	NM_017372.3
Muc2	ATGCCCACCTCCTCAAAGAC	GTAGTTTCCGTTGGAACAGTGAA	NM_023566.4
Itln1	TGACAATGGTCCAGCATTACC	ACGGGGTTACCTTCTGGGA	XM_029475723.1
Retnlb	AAGCCTACACTGTGTTTCCTTTT	GCTTCCTTGATCCTTTGATCCAC	XM_021185515.1
Ang4	GGTTGTGATTCCTCCAACTCTG	CTGAAGTTTTCTCCATAAGGGCT	XM_021154346.1
Tnf-a	GCACAGAAAGCATGATCCGC	CCCCATCTTTTGGGGGAGTG	XM_021218154.1
Il-6	CACTTCACAAGTCGGAGGCT	CTGCAAGTGCATCATCGTTGT	XM_021163844.1
Il-1b	TCTTTGAAGTTGACGGACCC	TGAGTGATACTGCCTGCCTG	XM_006498795.5
Mcp-1/Ccl2	CAGGTCCCTGTCATGCTTCT	TCTGGACCCATTCCTTCTTG	NM_011333.3
Il-10	GCTCTTACTGACTGGCATGAG	CGCAGCTCTAGGAGCATGTG	XM_036162094.1
Il-17a	TGAGCTTCCCAGATCACAGA	TCCAGAAGGCCCTCAGACTA	XM_021176489.1

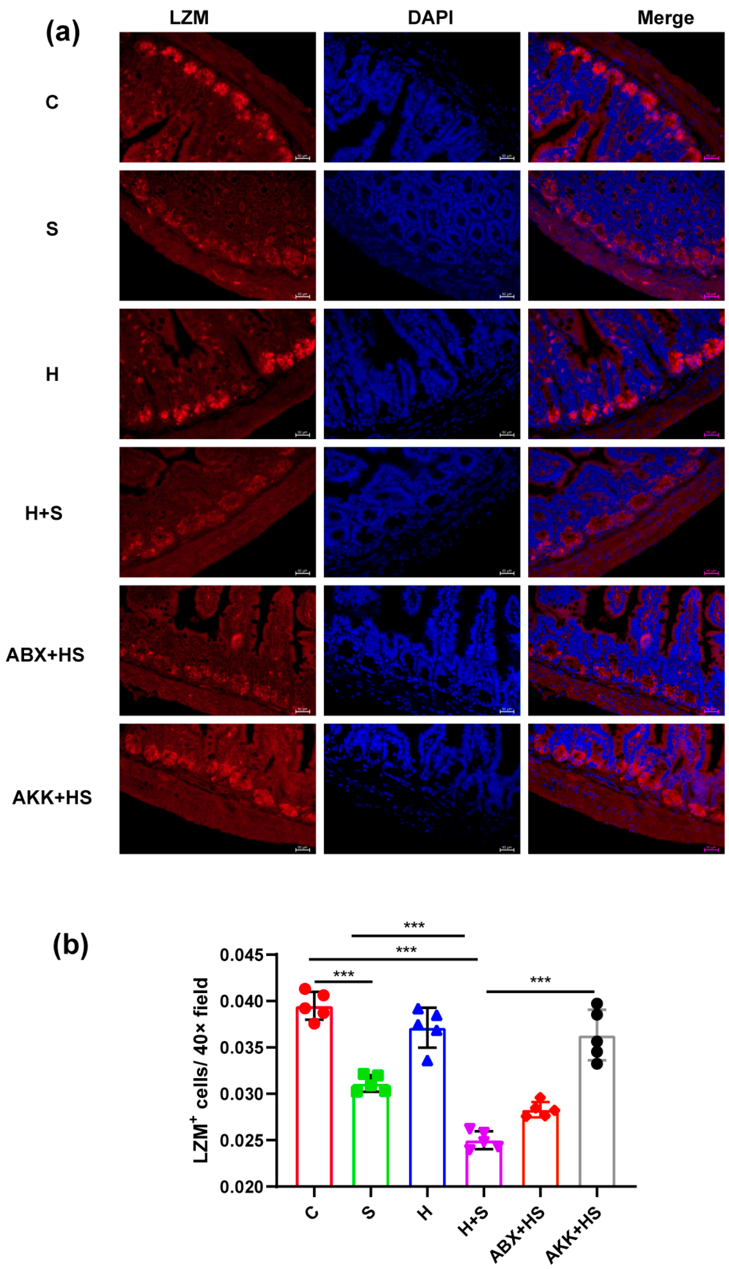

Supplementary Figure S1. Changes of jejunal Paneth cells after *A. muciniphila* colonization in mice stimulated by high fructose and restraint stress

a Immunofluorescence staining of jejunal Paneth cells (LZM, red) and nucleic acid (DAPI, blue) in the C, S, H, H+S, ABX+HS and AKK+HS groups. (n=5) Bar represents 50 μm. b Quantification of the Paneth cells in jejunal crypts. Data are presented as the mean ± SEM. ***$P < 0.001$ compared indicate significant difference. C: control group; S: restraint stress; H: high-fructose; H+S: high-fructose and restraint stress. ABX+HS: Antibiotic treatment + high fructose and restraint stress group; AKK+HS: *A. muciniphila* colonization + high fructose and restraint stress group.

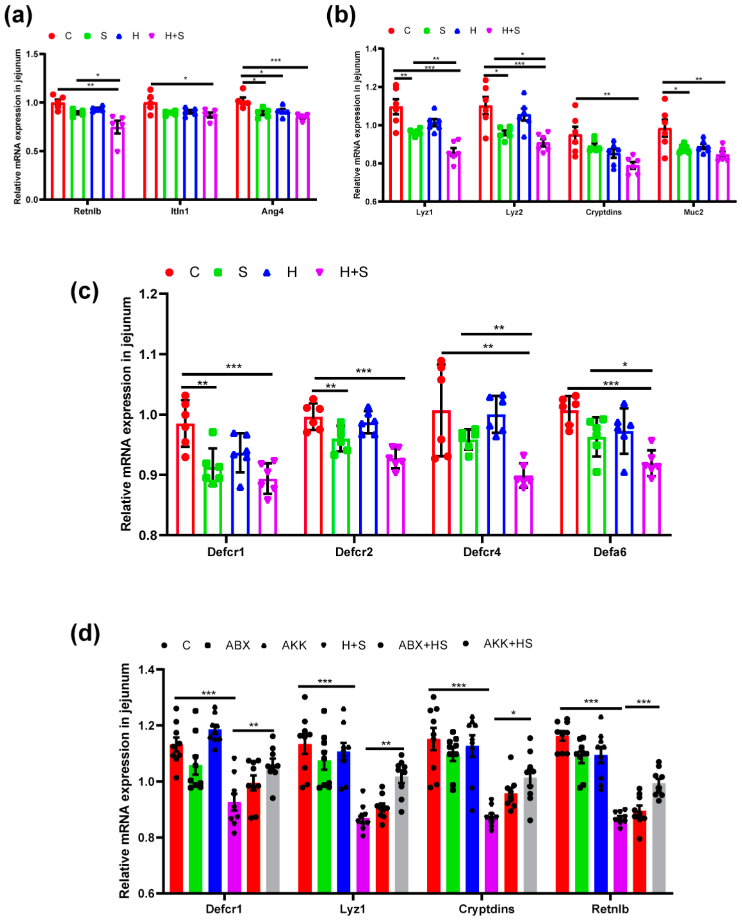

Supplementary Figure S2. Effects of *A. muciniphila* colonization on the expression of jejunal antimicrobial peptides in high fructose and stress-stimulated mice

a Changes in mRNA levels of Retnlb, Itln1, Ang4 in jejunum tissue (n=9). Changes in mRNA levels of Lyz1, Lyz2, Cryptdins, Muc2, Defcr1, Defcr2, Defcr4 and Defa6 in jejunum tissue(n=9) (b-c). d Changes of mRNA levels of antimicrobial peptides Defcr1, Lyz1, Cryptdins, Retnlb (n=9). Data are presented as the mean ± SEM. *P < 0.05; **P < 0.01; ***P < 0.001 compared indicate significant difference. C: control group; S: restraint stress; H: high-fructose; H+S: high-fructose and restraint stress. ABX: Antibiotic treatment group; AKK: *A. muciniphila* colonization group; ABX+HS: Antibiotic treatment + high fructose and restraint stress group; AKK+HS: *A. muciniphila* colonization + high fructose and restraint stress group.
